# Physical rehabilitation delivery by community health workers: Views of the users and caregivers

**DOI:** 10.4102/phcfm.v17i1.4852

**Published:** 2025-04-30

**Authors:** Miriam Mapulanga, Thembelihle Dlungwane

**Affiliations:** 1 Discipline of Public Health Medicine, School of Nursing and Public Health, University of KwaZulu-Natal, Durban, South Africa

**Keywords:** users and caregivers, experiences and perspectives, physical rehabilitation services, community health workers, Lusaka

## Abstract

**Background:**

Community health workers are crucial in providing health services at the community level. However, in Zambia, there are limited opportunities for formal physical rehabilitation training for community health workers, leading some to provide rehabilitation services without the necessary training.

**Aim:**

This study sought to explore the experiences and perspectives of users and caregivers who receive physical rehabilitation services from community health workers without training in physical rehabilitation.

**Setting:**

Matero, a sub-district of Lusaka, Zambia.

**Methods:**

An exploratory qualitative approach using face-to-face, in-depth interviews was used to collect data from users and caregivers who receive physical rehabilitation services from untrained community health workers. The study included 12 participants, six service users and six primary caregivers. Data were transcribed and analysed using thematic analysis.

**Results:**

Six themes emerged from the analysis, namely access to services, perceived skills and competence of community health workers, satisfaction with community health worker-delivered physical rehabilitation services, values and attitudes of community health workers, perceived unmet needs of community health worker-delivered physical rehabilitation services and impact of community health worker-delivered physical rehabilitation.

**Conclusion:**

Physical rehabilitation rendered by community health workers positively impacted the users despite the gaps identified. Formalising training of community health workers in physical rehabilitation could enhance service coverage and improve the overall quality of care.

**Contribution:**

The scientific contribution has been made by the views of users and caregivers regarding their experiences and perspectives of community health workers-delivered physical rehabilitation services without training.

## Introduction

Physical rehabilitation, including physiotherapy, occupational therapy, speech therapy and prosthetics and orthotics, is crucial for optimising physical functioning and reducing disability, aligning with Sustainable Development Goal 3 in Zambia.^1,2^ As a result of human resources for health in physical rehabilitation shortage, physical rehabilitation services are not universal and still need to be made available in resource-constrained settings, including Zambia.^2,3,4^ The demand for physical rehabilitation services has increased with the shift from communicable to non-communicable diseases; however, coverage remains low because of a persistent shortage of human resources for health.^4,5,6^ To address the unmet needs in rehabilitation, health system strengthening aims to integrate rehabilitation services into universal health coverage by incorporating rehabilitation at all levels of care, including primary health care to reach those in need.^7^ The World Health Organisation promotes this through the primary health care strategy, which has proven to deliver cost-effective and equitable healthcare and incorporates community health workers.^8^

Community health workers, selected by their communities, are vital in providing health services at primary health care centres, community health centres and in patients’ homes.^9,10^ Despite existing for over a century without much recognition, there is renewed interest in community health workers because of healthcare worker shortages, particularly in low-income countries.^10^ Community health workers bridge gaps in human resources for health, contribute to the reduction of morbidity with mortality and improve access to healthcare, because of their cultural and linguistic alignment with the communities they serve.^11,12^

Through task shifting, community health workers have played roles in various health areas, including communicable diseases and maternal health.^13,14^ Evidence shows that they can also address the shortage of human resources for health in rehabilitation by providing physical rehabilitation services.^15,16^ Depending on their training, community health workers contribute to physical rehabilitation through assessment, case management, health education and community-health system linkages.^17^ However, in Zambia, there is no formal training for community health workers delivering physical rehabilitation services. Some community health workers render physical rehabilitation services without relevant training. The current study sought to explore the experiences and perspectives of users and caregivers who receive physical rehabilitation services from community health workers without training in physical rehabilitation services. This study is part of a PhD study that seeks to propose an appropriate training model for community health workers delivering physical rehabilitation services in Zambia.

## Materials and methods

### Study design

An exploratory qualitative study design was adopted to understand the users’ and caregivers’ experiences and perspectives towards physical rehabilitation services delivered by community health workers in the Matero sub-district of Lusaka, Zambia. Face-to-face in-depth interviews were conducted to examine the experiences and perspectives of users and caregivers receive care from community health workers who deliver physical services.

### Study setting

This study was conducted in the Matero sub-district of Lusaka. According to the Health Master Facility List, the Matero sub-district is serviced by three public facilities: a first-level hospital, an urban clinic and a health post.^18^ The first-level hospital offers health services, including critical care medicine, internal medicine, dental services, ophthalmology, obstetrics and gynaecology, laboratory services, psychiatry and mental health, imaging services, surgery and physiotherapy. The urban health centre offers general practice consultation and treatment, dental services, laboratory services, physiotherapy, mother and child health services, with maternity and public health services. The health post offers public health services, general practice consultation and treatment, with mother and child health services. Twelve private clinics support these public facilities. Regarding physical rehabilitation, only the first-level hospital and the urban clinic offer services in the form of physiotherapy, with the first-level hospital having eight physiotherapy providers. In comparison, the urban clinic has six physiotherapy providers.^18^

### Study population and sampling

Matero sub-district has approximately 310 community health workers.^19^ These community health workers work as health volunteers at the community level. While they are members of the neighbourhood health committee and assigned operational zones by the community health chairperson, they are not integrated into the health care system. Purposive sampling was used to select zones that had community health workers who deliver physical rehabilitation services, though untrained. Potential participants were identified by the community health chairperson and were recruited into the study by the researcher. Users are clients who were receiving physical rehabilitation services rendered by community health workers for at least 1 year and were included in the study. Caregivers were individuals who cared for clients who were receiving physical rehabilitation services rendered by community health workers but could not express themselves because of language and speech barriers or being children. The users and caregivers who were interviewed were based on their availability at the time of data collection. Both users and caregivers of the users receiving rehabilitation and attending these services were included in the study. In cases where the service users were able to express themselves, the interview was conducted with the users. For children or clients with language and speech barriers, caregivers were interviewed instead.

### Data collection

The interviews were conducted from April to May 2021. The interviews were guided by the interview schedule of experiences and perspectives of physical rehabilitation services delivered by community health workers. While the schedule was in English, interviews were conducted in both English and Nyanja, the *lingua franca* of Lusaka. The principal investigator, a native of Lusaka with Nyanja as the first language, conducted the interviews. Appointments with the participants were made physically by the principal investigator through visiting the participants’ homes. All interviews were conducted at the residence of the study participants. Before the commencement of the interviews, information sheets about the study with a detailed explanation of the study’s aims were given to the participants. A consent form was signed by the participants prior to the interview. Questions from the participants were solicited, and time was allowed to give answers. Participants were informed of their free participation in the study and could withdraw at any time without any consequences. The principal investigator conducted all interviews. Interviews were between 45 and 60 minutes long. The interviews were conducted until saturation and redundancy were reached. All the interviews were recorded using a digital recorder and stored computer with a password for 3 years.

### Data analysis and management

The interview data were transcribed verbatim. The transcriptions were done by the principal researcher (MM) and verified by the supervisor (TD). The completeness and quality of the transcripts were verified by employing the observational notes taken during the interviews. The researcher (MM) read the transcript of each participant’s interview numerous times to understand the phenomenon fully and then identify codes. A thematic analysis was implemented, and emergent themes and subthemes were derived through coding.

Credibility by data triangulation was achieved through individual in-depth interviews, field notes during the interviews and re-reading of transcripts. An audit trail ensured the dependability of the collected data. The principal investigator (MM) and the supervisor (TD) were peer reviewers of the individual in-depth interviews to ensure confirmability. Thematic analysis was done concurrently with data collection, and when a comparison of new data and existing data showed no significant new codes, the data collection was halted as data saturation and redundancy were reached. Codes were identified by the principal investigator and the supervisor. Data from the study are safely locked on the computer with a password for 3 years.

### Self-reflection and researcher attributes

Both MM and TD are human resources for health in physical rehabilitation and public health specialists. While MM is a professional trainer for human resources for health for physical rehabilitation, TD is a public health trainer and an academic researcher. Before data collection, both researchers were unknown to the study participants and were never involved in physical rehabilitation service delivery in the Matero sub-district.

### Ethical consideration

All the participants gave written informed consent prior to data collection. The University of KwaZulu-Natal Biomedical Research Ethics Committee approved the study (BREC 00000569/2019), and the Zambia National Health Research Authority gave permission to collect data in Zambia. Additionally, consent forms were signed by the participants before the interview, and they agreed that the data they provided could be used anonymously for research and publication purposes. The participants had the right to opt out of the study without repercussion. Throughout the entire study, confidentiality, anonymity and the principle of non-maleficence were ensured.

## Results

### Profile of the participants

#### The users

The users’ ages ranged from 25 to 80 years. Most of the participants in the users’ category were men. Two users were retired, and the remaining four were unemployed. Table 1 shows the users’ profiles.

**TABLE 1:** Profile of the users.

Participant category	Age group	Sex	Level of education	Employment status	Condition
User-1	30–35	Female	Grade 12	Unemployed	Stroke
User-2	65–70	Male	Tertiary	Retired	Potts disease
User-3	25–30	Male	Grade 10	Unemployed	Spinal cord injury
User-4	75–80	Female	Tertiary	Retired	Stroke
User-5	50–55	Male	Tertiary	Unemployed	Stroke
User-6	55–60	Male	Grade 9	Unemployed	Stroke

#### The caregivers

The caregivers’ age group ranged from 50 to 80 years. All the caregivers were women, and most of them (*n* = 5) were unemployed. Table 2 shows their profiles.

**TABLE 2:** Profile of the caregivers.

Participant category	Age group	Sex	Level of education	Employment status	Relationship with the user	User’s age in years	User’s condition
Caregiver-1	75–80	Female	Tertiary	Retired	Mother	42	Stroke
Caregiver-2	65–70	Female	Grade 7	Unemployed	Wife	68	Stroke
Caregiver-3	70–75	Female	Grade 7	Unemployed	Sister	64	Stroke
Caregiver-4	50–55	Female	Grade 10	Unemployed	Mother	14	Cerebral palsy
Caregiver-5	65–70	Female	Grade 9	Unemployed	Grandmother	5	Cerebral palsy
Caregiver-6	50–55	Female	Grade 9	Unemployed	Wife	61	Stroke

### Emerging themes and sub-themes

A total of six themes emerged from the analysis of the exploration of users’ and caregivers’ experiences and perspectives towards physical rehabilitation services delivered by community health workers in Lusaka, Zambia. The emergent themes are as follows: (1) access to services, (2) perceived skills and competence of community health workers, (3) satisfaction with community health worker-delivered physical rehabilitation services, (4) values and attitudes of community health workers, (5) perceived unmet needs of community health worker-delivered physical rehabilitation services and (6) impact of community health worker-delivered physical rehabilitation services. Table 3 summarises the emergent themes and sub-themes.

**TABLE 3:** Themes and sub-themes.

Emerging themes	Sub-themes
Accessibility of services	Inadequate hospital sessions: time and attention not enough Inaccessible health facilities Enhance comfort and reduce anxiety associated with treatment
Perceived skills and competence of community health workers	Inadequate insight into certain conditions Insufficient hands-on experience Joint knowledge creation
Satisfaction with community health workers delivered physical rehabilitation services	Supportive and considerate Attentive and empathetic
Values and attitudes of community health workers	Respect and trust Receptive Cooperative
Perceived unmet needs of community health workers delivered physical rehabilitation services	Long waiting list Lack of partnership with health facilities
Impact of community health worker-delivered physical rehabilitation services	Physical improvement Improved activities of daily living Personalised care and tailored treatment Home context

### Access of services

The participants experienced various difficulties in accessing physical rehabilitation services at health facilities. The difficulties made the services inaccessible, so they preferred to use community health workers who delivered physical rehabilitation services at home. The following sub-themes emerged in the accessibility of health facilities for physical rehabilitation services: inadequate hospital sessions, inaccessible health facilities and lack of relaxation.

#### Inadequate hospital sessions: Time and attention not enough

Some participants felt that they required more and longer sessions in physical rehabilitation services than what was provided by the hospital and therefore sought services provided by the community health workers:

‘… at the hospital, when you join the session, there are a lot of children to be attended to, and sometimes yours may not even be attended to because of crying or he falls asleep. Moreover, if he is attended to, it may only be for a short period, like 10 minutes. Because of all this, I figured it is better to receive the services from a community health worker at home because here when she comes, she spends a good one hour with him and the child is given exclusive attention.’ (Caregiver 5, grandmother to 5-year-old cerebral palsy child)

‘We still attend physiotherapy at the clinic but it’s not enough. The time given to the patients is not enough. They teach us what to do at home quite alright, but it is not enough.’ (Caregiver 3, sister to 64-year-old stroke victim)

‘At the clinic, you cannot be attended to for thirty minutes because there are other people who are waiting to be attended to as well.’ (User 2, 65–70 years, Pott’s disease)

#### Inaccessible health facilities

The participants reported challenges that prevented them from going to the health facilities. Logistics because of distance was reported by some participants as the challenge as they could not afford to go to the hospital:

‘I cannot manage to go to the hospital because of distance. The service offered by the community health workers is convenient because it is offered at home.’ (User 1, 30–35 years old female stroke victim)

‘The clinic is quite a distance from here. So there is a need for a transport budget, which I cannot afford but the community health worker comes right here.’ (User 6, 55–60 years old male stroke victim)

‘Even though services are free at the clinic, the distance involved and my state means that I need to book a cab just [*to*] go and come back. We do not have money for the cab.’ (User 2, 65–70 years old male, Pott’s disease)

#### Enhance comfort and reduce anxiety associated with treatment

Uncomfortable situations resulting in lack of relaxation, making it difficult to exercise, were also reported as a reason for using community health worker-delivered physical rehabilitation:

‘Whenever we go to the clinic, his blood pressure goes up; I am unsure whether it is fear or anxiety. While we understand that the community health workers are not as good as the health providers, we had to opt for her services offered here at home because of that.’ (Caregiver 2, wife to 68 years old stroke victim)

‘My grandson cries too much at the clinic so there is no time for him to be touched by the provider. He is calm at home.’ (Caregiver 5, grandmother to 5-year-old cerebral palsy child)

### Perceived skills of community health workers

The participants shared their perspectives regarding the skills of community health workers in physical rehabilitation. The perspectives emanating from their experiences with community health workers’ service delivery fall into the following domains: inadequate insight about specific conditions, insufficient hands-on experience and joint knowledge creation.

#### Inadequate insight into certain conditions

The participants complained about the community health workers’ lack of information about specific conditions in their delivery of physical rehabilitation services:

‘She does not know and understand my condition. In the beginning, I had to educate her about my condition. Although she was readily available to support me, she relied on me to supply the knowledge. I have benefited from the exercises she helps me with, but nothing about Tuberculosis itself.’ (User 2, 65–70 years old male with Pott’s disease)

‘She does not understand my condition. With a raised blood pressure, she still insists that we do the exercises. Her help ends at practice. She has no theoretical explanation to give. If you need any theoretical explanation, you will need to go back to the clinic.’ (User 3, 25–30 years old male with Spinal Cord Injury)

#### Insufficient hands-on experience

The lack of information regarding certain conditions among community health workers was reflected in the performance and approach to treatment. The lack of practical skills by the community health workers led to an improper approach to case management, with different cases viewed as the same. According to the participants, community health workers need to gain skills in making gadgets and providing communication support:

‘I wish she could learn how to help the children with other things like the special chair. If she did this, it would be very useful and cheaper for me. She does not know how to use gadgets.’ (Caregiver 5, grandmother to 5-year-old cerebral palsy child)

‘The communication training to indicate when he wants something, has not been good. I would want to train him to look at a person directly whenever he wants something since he can hold his head. However, there is not much help with communication so far. She said she does not know how to do it for him.’ (Caregiver 2, wife to 68-year-old stroke victim)

#### Joint knowledge creation

The community health workers’ lack of insight into certain conditions led to joint knowledge creation. This led to collaborative efforts between the community health worker and users to create innovative methods of care and rehabilitation:

‘She does not know and understand my condition. In the beginning, I had to educate her about my condition. Although she was readily available to support me, she relied on me to supply the knowledge.’ (User 2, 65–70 years old male with Pott’s disease)

‘When the community health worker started having sessions with me, I had to explain to her that making me stand is a slower process because when it is done suddenly, I feel dizzy. This time she understands and she [*is*] patient when helping me to stand … she does not understand my condition. With a raised blood pressure, she still insists that we do the exercises. Her help ends at practice. She has no theoretical explanation to give. If you need any theoretical explanation, you will need to go back to the clinic.’ (User 3, 25–30 years old male with Spinal Cord Injury)

### Satisfaction with community health workers-delivered physical rehabilitation services

The community health workers’ manner of service delivery, such as being supportive, considerate, attentive and helpful and using the natural environment for exercises, satisfied the participants.

#### Supportive and considerate

The participants were satisfied with the community health workers’ support and consideration. The humane feelings of the community health workers towards their clients led to service satisfaction by some participants:

‘When my child was having feeding issues and getting choked easily, the community health worker escorted me to the hospital so that she could also learn how to feed him.’ (Caregiver 4, mother to 14-year-old cerebral palsy child)

‘I receive support when doing exercises for strength and it has been quite beneficial. This far, I can stand up without help from another person, and I can even move around the house. Of late, I go just outside the gate. Before I started training with her, I was being helped most of the time, I depended on my wife to escort me to the toilet. Now I can go to the toilet alone because I am stronger.’ (User 6, 55–60 years old male stroke victim)

‘The community health worker is considerate of my circumstances and understands the child’s limitation when it comes to his speech. She engages in talking to him as an individual he is and also acknowledges him even when she knows that he would not answer back.’ (Caregiver 5, grandmother to 5-year-old cerebral palsy child)

#### Attentive and empathetic

Some participants were satisfied with the attention and empathy shown by the community health workers in delivering physical rehabilitation services:

‘The good thing is that my child is given the attention since he is followed home. When the community health worker comes, I am assured that my child will be getting all the required attention because he is the only one here.’ (Caregiver 5, grandmother to 5-year-old cerebral palsy child)

‘The community health worker is very helpful to me. She helps me by offering support when doing exercises.’ (User 2, 65–70 years old male with Pott’s disease)

### Values and attitudes of community health workers

The participants in this study revealed the values and attitudes of community health workers delivering physical rehabilitation services, with the following sub-themes: respect and trust, receptive listening to cooperation support and support.

#### Respect and trust

It was revealed that the community health workers were respectful and trustworthy in their physical rehabilitation service delivery. One caregiver stated:

‘She is respectful in her communication with me and my son, it makes me happy.’ (Caregiver 4, mother to 14-year-old cerebral palsy child)

‘My information is safe with her. I have never heard it from another person. This makes me free to express my concerns to her, though I am much older than her. I have not known her to repeat my information elsewhere.’ (User 2, 65–70 years old male with Pott’s disease)

#### Receptive listening

In this study, the receptive attitude of the community health workers was revealed. It was revealed that the community health workers were receptive to their clients, making them comfortable. One user explained her experience:

‘I am very comfortable with her. I have known her for some time. I do express my concern and do not have problems expressing my special concerns.’ (User 2, 65–70 years old male with Pott’s disease)

#### Cooperative support

In this study, it was revealed that the community health workers’ physical rehabilitation service delivery was embedded in cooperation and support:

‘We work together. We want the same thing for my child, and we cooperate on many things. Where I have strengths, I share, and where she has, she shares. We collaborate on coming up with tasks to do.’ (Caregiver 5, grandmother to 5-year-old cerebral palsy child)

### Perceived unmet needs of community health workers-delivered physical rehabilitation services

Some participants perceived the long waiting list to access physical rehabilitation services as an unmet need, resulting in taking longer to access the services:

#### Long waiting list

The participants revealed that the community health workers’ other duties were the reason for the long waiting list. This was a result of the scheduling and appointments, which took longer:

‘After registering with the community health worker, it took a long time for her to start coming because she has many clients to visit.’ (User 3, 25–30 years old male with Spinal Cord Injury)

‘After I booked, it took a long time for her to start showing up because she has other duties.’ (User 6, 55–60 years old male stroke victim)

#### Lack of partnership with health facilities

Some participants pointed out the isolation of community health workers delivering physical rehabilitation services as the health facilities did not support them:

‘They do not work in partnership with the clinic.’ (User 6, 55–60 years old male stroke victim)

‘They are not known by the clinic so they cannot refer you. I just go to the clinic myself when I need to.’ (Caregiver2, wife to 68-year-old stroke victim)

### Impact of community health worker-delivered physical rehabilitation services

While the participants revealed that the community health workers lacked hands-on practical skills and knowledge for specific conditions, they revealed that their support enabled them to exercise from home. This improved the users’ quality of life despite the perceived unmet needs. The following sub-themes emerged: physical improvement, improved activities of daily living, personalised care and tailored treatment and home context.

#### Physical improvement

The clients’ improvement in physical strength was perceived as the impact of the community health workers’ support.

‘She has become stronger physically, she is able to engage in many activities. We are looking forward to seeing her go back to selling her merchandise at home or market.’ (Caregiver 3, sister to 64-year-old stroke victim)

‘With her support, I have made some improvements. I can do many things independently, I can tie my shoelaces and I can bath independently.’ (User 3, 25–30 years old male with Spinal Cord Injury)

‘The child has been improving slowly. He can now sit without support for about half an hour.’ (Caregiver 5, grandmother to 5-year-old cerebral palsy child)

‘From the exercises we have been doing, my daughter has now learnt to turn in bed by herself. She was not able to turn on her own until the community health worker started supporting me.’ (Caregiver 1, mother to 42-year-old stroke victim)

#### Improved activities of daily living

Some participants reported improvement in activities of daily living following the community health workers-delivered physical rehabilitation services because of the rehabilitation from home. A user expressed himself by saying:

‘The exercises incorporating home activities have been beneficial to me to my hand. I can now rinse dishes with both hands. I now need little help to bath. I have made some improvements.’ (User 1, 30–35 years old female stroke victim)

‘She helps me with exercises from here, my home. I have since been able to manoeuvre in the house with less help.’ (User 2, 65–70 years old male with Pott’s disease)

‘My hand can now grasp and function better. I am able to do dishes; at least I can now participate in household chores.’ (User 4, 75–80 years old female stroke victim)

#### Personalised care and tailored treatment

The participants highlighted that the community health workers’ visits provided them with personalised care and tailored treatment:

‘The progress is wholesome since enough time is allocated and home environment used.’ (Caregiver 6, wife to 61-year-old stroke victim)

‘Because it is at your home, the services are tailored to your home needs, which is good with continuity with life.’ (User 4, 75–80 years old female stroke victim)

‘My main problem is movement, so I should not be asked to go to the clinic. Furthermore, because the progress is slow, it helps to do the treatment at home.’ (User 4, 75–80 years old female stroke victim)

#### Home context

The participants appreciated the home-based services they received from the community health workers:

‘A person with disability should be taught how to navigate his environment; the hospital environment is not my environment, so even when I am taught things there, application matters. We need these people who visit homes.’ (User 4, 75–80 years old female stroke victim)

‘Home activities have been beneficial to me for my hand. I can now rinse dishes with both hands. I now need little help to bath. I have made some improvements.’ (User 1, 30–35 years old female stroke victim)

## Discussion

This study explored users’ and caregivers’ experiences and perspectives towards physical rehabilitation services delivered by untrained community health workers in Lusaka, Zambia. The participants in this study highlighted access to physical rehabilitation services at health facilities as one of the reasons for their use of community health workers-delivered physical rehabilitation. Inadequate hospital sessions and inaccessible health facilities were highlighted as significant drivers of users’ decisions to opt for community health worker-delivered rehabilitation. Previous studies have indicated that distance significantly influences rehabilitation service utilisation, compounded by financial constraints, as users struggle to pay their way to health facilities.^20,21^

The participants were satisfied with the support they received from the community health workers and emphasised that they were considerate and attentive. This humane manner of service delivery influences service acceptability by the community. This humane service delivery contributes to patients’ dignity, a prerequisite to quality health service delivery.^22^ This echoes what other studies have shown: with such positive attributes, community health workers are well placed to deliver culturally accepted services at the home level, including physical rehabilitation services.^23^

Community health workers improve service acceptability by embodying respect, trust, receptivity and cooperation. Respect, an individual’s moral right, is essential in healthcare to build meaningful relationships between providers and patients, promoting open communication.^24^ Trust is equally vital, correlating with higher service uptake and compliance.^25,26^ Equally, the community health workers’ trustworthiness enhances their role in community engagement, expanding access to services and supporting physical rehabilitation, thus widening service equity.^27^ Their cooperation, motivation and suitability for their roles demonstrate that community health workers could address gaps in primary health care in low-income countries like Zambia, where rehabilitation services are limited.^28^ Additionally, the values and ethics of the community health workers exhibited in this study form an important basis for skills development and could contribute to their training success.^29^

The participants said that they felt that the community health workers needed adequate insight into certain conditions. Furthermore, they revealed that the community health workers needed more hands-on practical experience. Admittedly, community health workers lack formal or paraprofessional education and usually depend on role-related training.^30^ However, rehabilitation-specific role-related training is necessary to acquire insight into conditions in physical rehabilitation and hands-on practical skills to deliver better physical rehabilitation services.^31^ Decentralised approaches to training community health workers, which include work-based learning and support in homes and at the clinic, have been demonstrated successfully.^32^ While the participants felt the community health workers needed adequate insight into certain conditions, the community health workers were aware of their lack of knowledge and skill. This provides an opportunity for in-service learning and clinical support focusing on individual cases.^33^

Long waiting times were flagged as barriers to users accessing community health worker-delivered physical rehabilitation services in this study. These delays largely stemmed from the high workload of community health workers, often tasked with multiple responsibilities.^34^ Aside from other health programmes to attend to, many community health workers also engage in other income-generating activities to support their families, reducing the time they can devote to health services.^35^ In addition to training in physical rehabilitation service delivery, in order to have a committed community health worker workforce, there is a need for structured employment that could provide a stable income for them to support their families.^36^ Financial incentives would further motivate community health workers, helping manage their workloads and enhancing community and home-level rehabilitation service delivery. Another significant finding is the need for stronger partnerships between community health workers and health facilities, as users often struggle with linkage to formal care. Coordinating care between community health workers and health facilities is critical, and meaningful partnerships would also support more seamless referrals.^37^ While integrating community health workers into the national healthcare system could foster these linkages allowing for supervision by trained health professionals, coordination of care is a local issue that can be addressed through the community health chairperson and the community health manager at the district level.

The fact that the community health workers provided service in the users’ homes was appreciated. In this study, home-based physical rehabilitation was deemed to enhance comfort and reduce anxiety associated with treatment. It was observed that the users’ activities of daily living were improved because of the use of home settings. These assertions align with study findings that suggest that individual personalised care in home contexts leads to better outcomes.^38,39^

While home-based services in this particular study were appreciated as health facilities are not easily accessible to the users because of movement limitations, rehabilitation care at home is important not just in resource-constrained settings but also in well-resourced settings.^38^ Rehabilitation aims for maximum functional recovery, with the home being the ideal setting for human function.

## Recommendations

Community health worker training in physical rehabilitation should encompass in-service training and clinical support. Local care coordination of the services should be designed and implemented to promote continuity of care and enhance referral systems between the community health workers and the health facilities. Community health workers delivering physical rehabilitation services should be integrated into the national health systems.

## Study limitations

This qualitative study was conducted in one district and may not be transferrable in other contexts. The study included participants who were available at the time of data collection and might not have captured the perceptions and experiences of the other clients.

## Conclusion

The findings from this study give insights into the user’s and caregivers’ experiences and perspectives of physical rehabilitation services delivered by untrained community health workers. This study suggests that community health workers positively impact the well-being of service users although they have limited skills because of a lack of training in physical rehabilitation. Training and integrating community health workers in physical rehabilitation could enhance service coverage, reduce inequalities and improve the quality of care.
